# *Salmonella* Serovars, Antibiotic Resistance, and Virulence Factors Isolated from Intestinal Content of Slaughtered Chickens and Ready-to-Eat Chicken Gizzards in the Ilorin Metropolis, Kwara State, Nigeria

**DOI:** 10.1155/2021/8872137

**Published:** 2021-03-02

**Authors:** M. A. Raji, H. M. Kazeem, K. A. Magyigbe, A. O. Ahmed, D. N. Lawal, I. A. Raufu

**Affiliations:** ^1^Department of Veterinary Microbiology, Faculty of Veterinary Medicine, University of Ilorin, Kwara State, Nigeria; ^2^Department of Veterinary Microbiology, Faculty of Veterinary Medicine, Ahmadu Bello University Zaria, Nigeria

## Abstract

Salmonellosis is one of the most common and widely distributed food-borne diseases, and the presence of antimicrobial-resistant *Salmonella* in poultry and poultry products is a global public health problem. Therefore, a cross-sectional study was conducted from November 2016 to July 2017 with an aim of determining the isolation rates of *Salmonella* species from the intestinal contents of slaughtered chickens, the most common serotypes that invade and colonize the tissues of chickens in Ilorin, and the susceptibilities of the isolated species to commonly used antibiotics. Four hundred samples of intestinal contents from apparently healthy slaughtered chickens and one hundred ready-to-eat chicken gizzards in Ilorin, Kwara State, were examined for the presence of *Salmonella* and their serotypes. *Salmonellae* were isolated and identified according to the techniques recommended by the World Health Organization: preenrichment, selective plating, biochemical testing, and serotyping. A total number of forty-three (43) *Salmonella* isolates consisting of 33 from intestinal contents and 10 from ready-to-eat chicken gizzards were isolated and identified. There was an overall *Salmonella* prevalence rate of 8.6% (43/500), and the isolates were distributed as follows: gizzard, 2% (*n* = 10) and intestinal contents, 6.6% (*n* = 33). The predominant serovars were *Salmonella enterica* subsp. *enterica* serovar 45: d: 1, 7 (16) and *S.* Haifa (5). All ready-to-eat chicken gizzards were associated with *Salmonella enterica* subsp. *enterica* serovar 45: d: 1, 7 (5). The *Salmonella* from intestinal contents belong to *Salmonella enterica* subsp. *enterica* serovars 45: d: 1, 7 (11) and *S*. Haifa (5). *Salmonella* species isolated were 100% resistant to ciprofloxacin, ampicillin, and ceftazidime. This is followed by cloxacillin (81%), tetracycline (75%), and sulfamethoxazole (67%). The *Salmonella* isolates were, however, 100% sensitive to enrofloxacin, 74% to streptomycin, and 72% to gentamycin antibiotics. The most common serotype was *S. enterica* subsp. *enteric*a serovar 45: d: 1, 7. All the twenty five *Salmonella* serovars consisting of twenty-one serotypes (*n* = 21), two of the *Salmonella* that could not be cultured after enrichment, and the two that were contaminated with *Proteus* possessed the virulence genes of *invA* and *stn*. The *Salmonella enterica* subsp. *enterica* serovar 45: d: 1, 7 and *S*. Haifa possess virulence genes so they are potentially virulent for humans in this area. The national and local health authorities in Nigeria should improve hygiene measures especially at retail slaughter markets to reduce salmonellosis which is one of the most important food-borne diseases in humans.

## 1. Introduction

Poultry meat and eggs are major sources of animal protein in Nigeria, as in many developing countries, because of their affordability and acceptance [1, 2]. This source is, however, being threatened by diseases such as salmonellosis and avian influenza [3]. Farmers still experience great losses (due to mortality, morbidity, and drop in egg production) caused by host-adapted *Salmonella* serovars despite huge amounts spent on vaccination and medication [2]. The industry has been facing devastating hazards; lack of disease control programs being one of the problems facing poultry production in Nigeria. Salmonellosis is a food-borne disease of primary concern in developed and developing countries. It is one of the major public health problems in terms of socioeconomic impact [1]. A wide array of animal reservoirs and commercial distribution of both animals and food products favor the spread of the disease [1–3].

Food-borne infections caused by *Salmonella* serotypes occur at high frequency in industrialized nations and developing countries and is an important public health problem worldwide [4]. In Nigeria, Typhimurium and Enteritidis are the two most common serotypes identified from different sources [2, 5, 6]. *Salmonella* serotype Enteritidis is currently the main cause of human salmonellosis in most industrial countries where human infections are generally associated with the consumption of contaminated food [7]. Because of its public health significance, salmonellosis has become one of the most important bacterial diseases affecting poultry. In the early sixties, *Salmonella* resistance to single antibiotics was reported, and since then, multiple drug resistance (MDR) has been reported worldwide [8, 9]. The global scenario has showed that there is an increased number of antibiotic-resistant *Salmonella* species from humans and farm animals [10, 11]. This resulted into a major public health concern that *Salmonella* species could become resistant to antibiotics used in human medicine thus reducing therapeutic options and threatening the lives of infected individuals. The uncontrolled use of antibiotics in farm animals and aquaculture systems has contributed tremendously to the emergence and persistence of resistant strains [12–14]. The situation of poultry-related food-borne illness in Nigeria is unknown, making it important for the need to conduct a survey of the prevalence of *Salmonella* serovars in poultry and poultry products in Ilorin, Kwara State. The present study was aimed at isolating and characterizing *Salmonella* species from chicken intestinal contents and ready-to-eat chicken gizzards by using enrichment and selective media and identifying these *Salmonella* species by conducting biochemical tests along with their antibiotic sensitivity patterns.

## 2. Materials and Methods

### 2.1. Study Area

Chicken intestinal contents were collected from two different live-bird markets. These markets include Oja Unity and Oja Ipata, all in the Ilorin metropolis of Kwara State, Nigeria. The chicken ready-to-eat gizzards were collected along Kwara Hotel in the Government Reserve Area (GRA) in Ilorin.

### 2.2. Description of the Study Area

**Figure 1:**
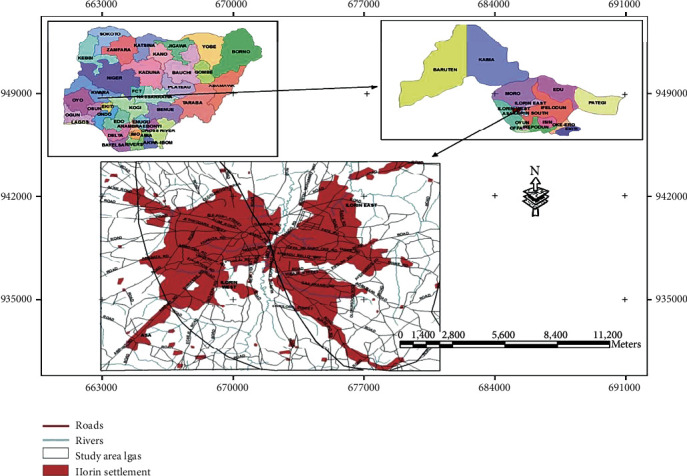
Map of the study area. Sources: Dooyum [15]. Isolation and antibiograms of *Staphylococcus aureus* of fresh cow milk and fried cheese from three local government units of Ilorin.

This study was conducted in Ilorin, Kwara State, Nigeria. Kwara State is located in the region termed the Middle Belt of Nigeria. It enjoys moderately dry and wet seasons, with heavier rain falling in September and October. It is within the forest savanna region of Nigeria. Kwara State lies between latitude 7°45N and 9°30N, longitude 2°30E and 6°23E. The state is bordered by the Oyo, Osun, and Ekiti states to the south, by the Kogi state to the east, and by the Benin Republic to the west. According to the 2016 national census, the Kwara State population was 2,871,089 people with a total area of 332,500 square kilometers or 8% of the land area of Nigeria. Ilorin is divided into three local government areas, namely, Ilorin East, Ilorin West, and Ilorin South (Figure 1) [15].

### 2.3. Study Design

A cross-sectional study was carried out to isolate *Salmonella* species from intestinal contents of slaughtered chickens and ready-to-eat chicken gizzards. Slaughter markets were used as the sampling frame with the markets being the sampling units. Samples that were collected included representative portions of intestinal chicken content during slaughter and ready-to-eat chicken gizzard. Two markets in Ilorin were randomly selected for this study and they include Oja Ipata and Oja Unity. The ready-to-eat chicken gizzards were collected from the Government Reserve Area (GRA) of the Ilorin metropolis.

### 2.4. Determination of Sample Size

A total of 400 chicken intestinal content swabs from live-bird markets during processing were collected and investigated for *Salmonella* and *Listeria* species during the period of September 2016 to April 2017. Sample size was calculated using the equation outlined by Thrusfield [16] by taking 11% prevalence rates of *Salmonella* species in raw chicken [6], where *n* = 1.962 × 0.115 (1 − 0.115) = 150.05238 = 150 samples.

### 2.5. Sample Size of Ready-to-Eat Chicken Gizzards

One hundred ready-to-eat chicken gizzard samples were also collected from a location in Suya (roasted and spiced chicken gizzard) in GRA, Ilorin, Nigeria, based on sample availability. However, a total of 500 samples were collected which comprised of 400 chicken intestinal contents and 100 samples from ready-to-eat chicken gizzards (samples were collected based on availability).

### 2.6. Sampling for *Salmonella* in Poultry

#### 2.6.1. Collection of Samples

Four hundred (400) samples of fresh intestinal feacal contents were obtained from apparently healthy chickens which have neither been diagnosed nor treated for *Salmonella* and were kept in domestic homes as free rangers or in poultry houses in the Ilorin metropolis, Kwara State, Nigeria; these samples were used for this study. The samples were collected immediately after the chickens were slaughtered; using a clean tweezer, they were placed inside separate sterile polythene bags and labeled. The samples were kept on ice until they reached the laboratory for analysis, as suggested by the International Organization for Standardization (ISO) [17].

#### 2.6.2. Analysis of Samples

Analysis of the intestinal contents was done in three phases: preenrichment, selective plating, and identification as described below.

#### 2.6.3. Preenrichment

One gram (0.5 g) of each of the intestinal contents was taken out aseptically and put in 4.5 ml of 0.1% peptone water (1 part to 9 parts peptone water) as suggested by the International Organization for Standardization (ISO) [17] The homogenized intestinal contents in peptone water were transported to the laboratory and incubated at 37°C for 48 hours.

#### 2.6.4. Selective Enrichment

One milliliter of each of the homogenized samples was transferred into selective plating

#### 2.6.5. Selective Plating

Plating was done using the procedures of Mebrat et al. [18]. Briefly, using a sterile wire loop, the broth cultures were inoculated onto *Salmonella* selective medium agar base plates (oxoid formulation) with xylose lysine deoxycholate agar (XLD) then incubated at 37°C for 48 hours under aerobic conditions. Typical colonies of *Salmonella* species were examined after 48 hours of incubation as recommended by Mebrat et al. [18].

The isolated colonies were identified on the basis of morphology, cultural characteristics, and their biochemical profile according to Cruickshank et al. [19].

#### 2.6.6. Gram's Staining

The test organisms were stained by Gram's method to determine their staining characteristics and purity of the culture. By this method, all isolates were observed for Gram negativity, shape, size, conformation, arrangement patterns, etc. Isolates of *Salmonella* were identified by IMViC reaction, TSI reaction, urease test, H_2_S production test, and nitrate reduction test as per methods described by Cruickshank et al. [19].

### 2.7. Serological Identification

All biochemically identified *Salmonella* isolates from examined sources were serotyped at the Thai National Institute of Health, *Salmonella* and *Shigella* Center, Department of Medical Sciences, Ministry of Public Health, Thailand. The serotyping was done by slide agglutination technique using polyvalent and monovalent antisera according to the Kauffmann-White scheme [20]. All the isolates of *Salmonella* strains were serotyped by using polyvalent O sera in the laboratory.

### 2.8. Antibiotic Susceptibility Testing

Antibiotic susceptibility testing was performed using the Kirby-Bauer method (disc diffusion technique) [21]. An inoculum was prepared with 3 to 4 colonies of pure culture onto nutrient agar (Mueller-Hinton agar) in a slope. These colonies were emulsified in a tube with 5 ml of physiological saline in order to obtain a homogeneous suspension with a density equivalent to 0.5 McFarland's standards. The discs used were manufactured by Oxoid Laboratories, UK. The sensitivity discs were specifically designed and contained appropriate concentrations of different Gram-negative antibiotics which include ciprofloxacin (10 *μ*g/disc), ampicillin (10 *μ*g), ceftazidime (30 *μ*g), ceftriaxone (30 *μ*g), gentamycin (500 *μ*g), streptomycin (10 *μ*g), sulphamethoxazole (300 *μ*g), tetracycline (30 *μ*g), nalidixic acid (30 *μ*g), cloxacillin (10 *μ*g), norfloroxacin (10 *μ*g/disc), gentamycin (10 *μ*g/disc), and streptomycin (30 *μ*g/disc). Both cultures of different isolates of the test organism were carefully swabbed on the surface of the Mueller-Hinton agar (previously prepared according to the manufacturer's instructions). The plates were incubated at 37°C for 48 hours. The different inhibition zone sizes were measured and recorded in millimeters (mm), and then the zone and size interpretive criteria of the National Committee for Clinical Laboratory Standards [22] were used to interpret the zone sizes. The strains resistant to three or more antimicrobials from different classes were considered as multidrug resistant (MDR).

### 2.9. The DNA Extraction Using Kit

The DNA extraction was done by a DNA extraction kit purchased from South Africa (Inqaba, South Africa). PCR was performed with two sets of primer pairs specific for the invasive gene *invA* and *stn* gene as shown in Table 1. PCR amplifications were performed in a final volume of 25 *μ*l containing DNA template (3 *μ*l), ×2 PCR Mastermix (MBI Fermentas) (12.5 *μ*l), 10 pmol/*μ*l of each primer (NG 2017-5571, Inqaba, South Africa) (1 *μ*l), and 5.5 *μ*l nuclease-free water. Amplification for the *invA* gene was carried out as described by Liu et al. in 2002 with minor modifications. The reaction conditions involved initial denaturation at 94°C for 3 min, followed by 35 cycles of 94°C for 30 s, 63°C for 30 s, and 72°C for 30 s. A final extension of 5 min at 72°C was employed. The amplification for the *stn* gene was carried out employing the same conditions as *invA* except annealing at 55°C. Amplification products were separated by electrophoresing on 2% agarose gel stained with 5 *μ*g/ml of ethidium bromide with a 100 bp DNA ladder as a molecular weight marker.

### 2.10. Data Management and Analysis

Data management, entry, and analysis were employed using Microsoft Office Excel 2007. Descriptive statistics such as percentage and proportion were used to describe samples detected positive to *Salmonella* isolation from the total sample analyzed by sources of samples and sample type. It was generated using the procedure of frequency (FREQ) and expressed in percent. Pearson's chi-square (*χ*^2^) test was used to determine the significance of difference or variation of prevalence. *P* value of less than 0.05 was considered to determine statistically significant differences. All statistical analysis was performed using the SPSS software package (version 15.0).

## 3. Results

### 3.1. Isolation Rates of *Salmonella* and *Listeria* Spp. in Chicken Intestinal Contents in the Ilorin Metropolis

In this study, 43 (8.6%) of the 500 samples were found to be positive for *Salmonella* species, and among them, 33 (6.6%) and 10 (2%) intestinal content and ready-to-eat chicken gizzard samples were positive with *Salmonella* species, respectively (Table 2). There was a significant difference between contaminated intestinal content samples and ready-to-eat chicken gizzard samples (*P* < 0.05). There is no significant difference between different markets examined in this study. Listeria was isolated from this study based on colonial morphology and Gram stain reaction. Most of the isolates were gram-negative rods. The percentage isolation from intestinal contents from Oja Unity and Oja Ipata showed 4.4% and 2.2%, respectively, out of the total of 400 samples collected. *Salmonella* species isolated based on breed showed that broiler accounted for 10.4% of *Salmonella* from Oja Unity (7.32%) and Oja Ipata (3.1%). *Salmonella* species were not isolated from local chicken and cockerel chicken in this study. The results further demonstrated that an overall isolation rate of *Salmonella* from layers and broilers were 7.3% and 10.4%, respectively. The values of layers in Oja Unity were comparably higher (4.59%) than those in Oja Ipata (2.75%). There was a difference in the value of broilers in Oja Unity (7.32%) compared with that in Oja Ipata (3.1%) (Table 3).

### 3.2. Antimicrobial Resistance Pattern of *Salmonella* Species Isolated from Intestinal Contents and Ready-to-Eat Chicken Gizzards

The resistance profiles of *Salmonella* species to 10 antimicrobial agents tested in this study are shown in Table 4. Forty-three (100%) out of 43 isolates of *Salmonella* species were resistant to more than 1 antibiotic agent. Total resistance (100%) to ciprofloxacin, ampicillin, and ceftazidime was obtained in this study, followed by cloxacillin (81%) tetracycline (75%), and sulfamethoxazole (67%). The *Salmonella* isolates were, however, 100% sensitive to enrofloxacin, 74% to streptomycin, and 72% to gentamycin. The isolates from ready-to-eat chicken gizzards were particularly resistant to ciprofloxacin, tetracycline, nalidixic acids, ampicillin, cloxacillin, and sulphathiazoles. The isolates were resistant to multidrugs especially quinolone, cycline, and the B-lactamase group of antibiotics. Resistance to multidrugs was observed in this study from resistance to a minimum of three classes of antibiotics to a resistance to a maximum of six classes of antibiotics (Table 5).

### 3.3. Occurrence of *Salmonella* Serotypes in Intestinal Contents and Ready-to-Eat Chicken Gizzards in the Ilorin Metropolis

A total of forty-three isolates of *Salmonella* were sent for serotyping in Thailand. Twenty-one isolates were serotyped. Eleven isolates were unable to grow after they were enriched in broth at Thailand which may be due to transportation stress. Eleven of the *Salmonella* isolates were contaminated with *Proteus* which could not be serotyped. The top serotypes identified in this study were *Salmonella enterica* subsp. *enterica* serovar 45: d: 1, 7 (*n* = 16) which accounted for 37.21% of the isolates, followed by *S.* Haifa (*n* = 5) which accounted for 11.63%. The serotypes from intestinal contents were *S. enterica* subsp. *enterica* 45: d: 1, 7 and *S.* Haifa. Only *Salmonella enterica* subsp. *enterica* 45: d: 1, 7 was obtained from ready-to-eat chicken gizzards. Serotype prevalence and distribution in chicken intestinal contents and ready-to-eat chicken gizzard samples are reported in Table 6.

## 4. Discussion

In this study, 8.6% of the intestinal contents and ready-to-eat chicken gizzards were positive for *Salmonella* species. This implies that apparently healthy adult chickens are carriers of *Salmonella* in Ilorin. The overall prevalence rate of 8.6% obtained in this study was close to the 10.8% obtained by Agada et al. [24] from poultry and humans in Jos, Nigeria. Another study in Ibadan by Fashae et al. [6], prevalence rates of 11% from chicken faecal samples were reported in their study. High isolation rates of *Salmonella* have been reported by Rauf et al. [25], who reported a prevalence of 2 to 16% from three poultry slaughter houses and five intensively managed poultry farms in a circumscribed area of Maiduguri, Nigeria. The result of this finding is different from Abdoulaye [5] who reported 15% prevalence rates of *Salmonella* from apparently healthy local chickens sold and slaughtered at a retail market in Zaria. Fagbamila et al. [2] also found high (43.6%) *Salmonella* prevalence rates among commercial poultry farms in Nigeria with state prevalence ranging from 11.1 to 65.4%. Ameh et al. [26] also reported high prevalence rates of *Salmonella* in chicken meat in Maiduguri as high as 27%. The findings in this study disagreed with previous studies conducted outside Nigeria by Selvaraj et al. [27], who reported lower isolation rates of *Salmonella* species from intestinal contents of chickens (5.26%) in India. From the same study, *Salmonella* was also isolated from kidneys and gizzards (3.57%). Traore [28] reported a contamination level of *Salmonella* of 55.66% in chicken intestines in Cȏte d′Ivoire but no data was available concerning the contamination rates of *Salmonella* in chicken gizzards in that study. Similarly, a high prevalence rate of 67% was reported by Dione et al. [29] in Gambia from chicken faecal samples. The result of *Salmonella* isolation rates of 2% from ready-to-eat chicken gizzards disagreed with that of Abdel-Aziz [30] who reported the prevalence of *Salmonella* from gizzards in Egypt to be 6.6%. The findings of the present study disagreed with those of Cardinale et al. [31] who reported a 43.3% prevalence of *Salmonella* species from raw gizzards in Senegal. In Ethiopia, 53.1% isolation rates of *Salmonella* species were reported by Tibaijuka et al. [32]. In 2003, another report was made by Traore from Abidjan, Cȏte d′Ivoire showing that braised chicken gizzards are contaminated with *Salmonella* at rates of 3.33%. Another study in Spain by Capital et al. in 2003 also reported that 55% of the carcasses and 40% of the giblets (gizzards and livers) were contaminated with the *Salmonella* species; this was higher than the findings in this study.*Salmonella* organisms were implicated as major causes of microbial food spoilage and contamination of ready-to-eat chickens [33, 34]. They may constitute an important source for a spread in the environment. The difference between our results and those of other findings may be due to differences in the hygienic status of each location where the samples of chickens were collected, the types of organ from which samples were collected, the methods of isolation, the culture media used, and environmental factors.

There were more *Salmonella* isolated in broilers (10.4%) than in layers (7.3%). The results show that there were more from Oja Unity than in Oja Ipata. High prevalence rates (37%) of *Salmonella* contamination of broiler farms have been reported from Algeria by Elgroud et al. [35]. High prevalence rates of *Salmonella* species have been reported by Ishihara et al. [36] who reported rates of 36% in broiler faecal samples in Japan. Barua et al. [37] also reported high prevalence rates (18%) of *Salmonella* from broilers in Bangladesh. The results of this study contradict the findings of Dione et al. [29] in Gambia who reported 67% of *Salmonella* isolation in laying birds in their study. Similar high prevalence rates of *Salmonella* (42%) were reported by Tabo et al. [38] in NDjamena, Chad, from laying hen flocks. High isolation rates of *Salmonella* have been reported also in Ghana by Andoh et al. [39]. The presence of *Salmonella* in intestinal contents could be related to the asymptomatic carrier status of some chickens that continue to shed *Salmonella* without showing any clinical signs [5, 6]. This could result in contaminated animals for slaughter, which poses a risk of transfer on carcasses. The carcasses could have been contaminated during removal of feathers or during evisceration.

In this study, the *Salmonella* isolates from chicken intestinal contents and ready-to-eat chicken gizzards were resistant (100%) to ciprofloxacin, ampicillin, and ceftazidime followed by cloxacillin (81%), tetracycline (77%), nalidixic acid (56%), and sulfamethoxazole (67%). The results of the present study agreed with the observation of Agada et al. [24] who also reported that *Salmonella* isolated from poultry in Jos were resistant to ampicillin (96%), ceftazidime (84%), and to oxytetracycline (63%). The results of this study also agreed with those of Fashae et al. [6] who reported that *Salmonella* isolated from poultry in Ibadan was highly resistant to tetracycline (93%), nalidixic acid (81%), and sulphamethoxazole (87%). Another study conducted in Nigeria and India by Adesiji et al. [40] has also shown the resistance of *Salmonella* isolates from poultry and human sources to tetracycline (66.7%) and nalidixic acid (60%). The susceptibility testing results showed that the *Salmonella* isolates tested were sensitive to enrofloxacin (100%), streptomycin (74%), and gentamicin (72%). The resistance to ciprofloxacin is consistent with the prevalence of 92-96% reported from Nigeria by Raufu et al. [41]. This result also disagreed with Fashae et al. [6] who reported 3% resistance to ciprofloxacin in their study in Ibadan, Nigeria. Agada et al. [24] also reported 100% sensitivity to ciprofloxacin in Jos, Nigeria. The high prevalence of nalidixic acid resistance among poultry isolates (66%) was also reported from France in 2000 [42]. Resistance to trimethoprim-sulfamethoxazole among poultry isolates was reported from Senegal [43], Mexico [44], and the USA [45]. Among the fluoroquinolones, resistance to ciprofloxacin was found to be comparatively highest in the present study as compared to 35% resistance in the USA [46], 10.2 to 16.8% in Germany [47], and 9.6% in Austria [48]. The isolates showed the highest antibiotic sensitivity to enrofloxacin (100.00% sensitivity) which was in correlation to the reports of Zahrei et al. [49]. Most *Salmonella* isolates (77%) in this study were resistant to tetracycline. Tetracycline resistance among food production animals has been attributed to selection pressure exerted from diverse sources such as prophylaxis, veterinary therapy, and use of antibiotics for animal growth promotion [50, 51]. The mechanisms of antimicrobial resistance may be broadly divided into genetic and phenotypic. Genetic resistance may be because of chromosomal mutation or acquired genes that are harboured on transposons or plasmids [51]. Tetracycline resistance may occur through tetracycline modification, ribosome protection, and tetracycline efflux [51]. Therefore, resistance to drugs such as oxytetracycline could be expected since the members of this class (tetracycline and chlortetracycline) are approved for use in broiler feeds for the purpose of growth promotion [51]. Although the frequency of resistance is high, continuous surveillance is important to monitor the emergency of antibiotic resistance of *Salmonella* strains.

The demonstration that meat products are a source of antibiotic-resistant *Salmonella* strains is a serious concern for public health and food safety. The widespread overuse and misuse of antimicrobial agents are associated with the development of resistance to these drugs that has emerged as a major problem worldwide [45]. The possibility that antimicrobial-resistant bacteria may be transferred to humans through the food chain and the possibility that the selection of novel antimicrobial resistance mechanisms in *Salmonella* in animals may specify resistance to antibiotics used in humans are a cause of concern [6]. The current study indicated the necessity for further investigation on the molecular characterization of the isolates with emphasis on resistant strains which is also necessary for identifying the mechanisms of antibiotic resistance.

The most prevalent *Salmonella* serovar in this study was *S. enterica* subsp. *enterica* serovar 45: d: 1, 7 (37.21% of the isolates). This result was consistently similar to results reported in other studies [5, 6, 52]. Numerous *Salmonella* serotypes are pathogenic. This includes *S*. *enterica* serovar *enterica* and *S*. Haifa, which have been reported in Nigeria by Fashae et al. [6] and Abdoulaye [5]. The most common serotype identified in the present study was *S*. *enterica* subsp. *enterica* serovars 45: d: 1, 7 (36.21%). Raufu et al. [41] identified a predominant serotype of *Salmonella* Hiduddify from free-range chicken and poultry meat in his study which was not isolated in this study. It may be that the birds Raufu et al. [41] examined are local free-range chickens as opposed to chickens from intensively managed chicken farms. Our results were consistent with investigation from the intensively managed chicken farms in Nigeria and in the Sichuan areas of China were serotype *S*. Haifa and other serotypes were identified [6, 53]. In another study by Agada et al. [24] in Nigeria and in a study by Selvaraj et al. in India [27], they were not able to isolate and identify the serotypes found in this study in their works. But the most common isolated *Salmonella* from the intensively managed chicken farms in Cambodia, Vietnam, and South Korea were *S*. *enterica* serovar Anatum, *S*. *enterica* serovar Infantis, and *S*. *enterica* serovar Hadar, respectively [53, 54]. In Nigeria, however, there is a paucity of such reports both in *Salmonella* serotypes from human and food animal origins. The difference of the *Salmonella* serotype distribution may mainly be related with area differences. *Salmonella enterica* subsp. *enterica* is a common cause of human gastroenteritis and bacteraemia worldwide ([55–58]). A wide variety of animals, particularly food animals, have been identified as reservoirs for non-Typhi *Salmonella* [59–61]. Although approximately 2,600 serovars of *Salmonella enterica* have been identified, most human infections are caused by a limited number of serovars, and in general, these infections are self-limited. When compared to other serovars of non-Typhi *Salmonella*, infections with these serovars are associated with higher rates of bacteraemia, meningitis, and mortality [55, 62–65].

**Figure 2:**
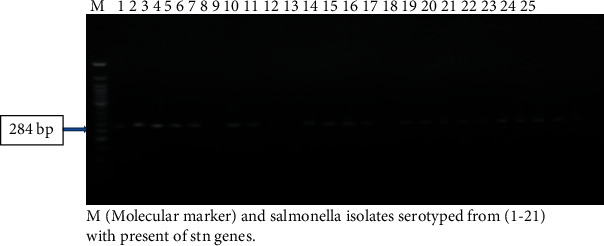
Amplification of the *stn* gene from *Salmonella* serovars from intestinal contents and ready-to-eat chicken gizzards from the Ilorin metropolis.

**Figure 3:**
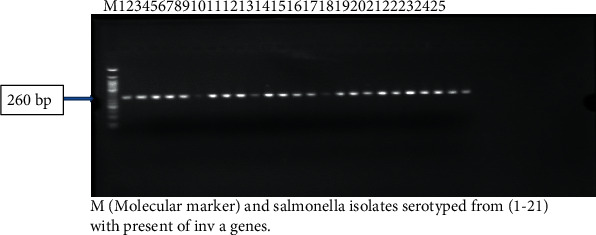
Amplification of *invA* gene from *Salmonella* serovars from intestinal contents and ready-to-eat chicken gizzards in the Ilorin metropolis.

All 25 *Salmonella* isolates (16 of the isolates belong to *Salmonella enterica* subsp. *enterica* and 5 isolates belong to *S.* Haifa, see Table 7; also included in the molecular study are two isolates that could not grow after enrichment in Thailand and two of the *Salmonella* isolates that were contaminated with *Proteus*) were examined for *invA* and *stn* genes by PCR. In the present study, the *stn* gene was detected in 100% and 98% of *S. enterica* subsp. *enterica* serovars 45: d: 1, 7 and *S.* Haifa in Ilorin, Nigeria, respectively. Studies have reported similar results [66–68] indicating that the *inv* A gene is present in most *Salmonella* serotypes which is expected since *invA* is an invasive gene conserved among the *Salmonella* serotypes. Electrophoreses results of the *invA* and *stn* genes are shown in Figures 2 and 3. *Salmonella*-induced diarrhoea is a complex phenomenon involving several pathogenic mechanisms including production of enterotoxin [67]. This enterotoxin production is mediated by the *stn* gene [67]. This *stn* gene has been reported to be absent in *S. bongori* [69] strains and also the other members of *Enteriobacteriaceae* or *Vibrio*, which have enterotoxigenic potential [70]. In India, the *stn* gene was, respectively, detected in 81.2 and 78.4% of *S.* Typhi and *S.* Paratyphi A but not in *S.* Typhimurium isolated from humans [70]. However, Murungkar et al. [71] detected the *stn* gene in all *Salmonella* isolates from five different serovars and four different sources. Thus, all the *Salmonella* isolates were found highly invasive and enterotoxigenic. The presence of the *stn* gene in all the clinical isolates highlights the role of the *stn* gene in the production of enterotoxin, which is responsible for causing acute gastroenteritis. The negative isolates may have lost the gene during their evolution. Studies concerning the frequency of these genes are important in tracking the adaptation of different serovars of *Salmonella* spp. to an increasing number of hosts [72]. Although it is not possible to predict whether a particular serovar of *Salmonella* will cause the disease merely by the presence or absence of a few virulence genes, the high prevalence of multiple virulence genes from the isolates could explain the increased potential of the serovar in causing severe infections in humans in Ilorin, Kwara State. In conclusion, this study revealed the prevalence of various *Salmonella* serovars and the emergence of multiple drug-resistant *Salmonella* serovars from chicken intestinal contents and ready-to-eat chicken gizzards in Ilorin, Nigeria. Prudent use of antibiotics is essential, and its continuous use as a growth promoter might need to be reexamined.

## Figures and Tables

**Table 1:** Primer sequence and primer size used in this study.

*invA*	F: GTG AAA TTA TCG CCA CGT TCG GGC AA	284 bp
	R: TCA TCG CAC CGT CAA AGG AAC C	
*Stn*	F: CTT TGG TCG TAA AAT AAG GCG	260 bp
	R: TGC CCA AAG CAG AGA GAT TC55	

Source: Liu et al. [23], antimicrobial resistance and resistance genes in *Salmonella* isolates from chicken.

**Table 2:** *Salmonella* isolation from the intestinal content samples from Oja Unity and Oja Ipata and ready-to-eat chicken gizzard samples in the Ilorin metropolis.

Market	Number of chickens and ready-to-eat gizzards tested	Number of chickens and gizzards that were positive	Percentage of positives per sample type
Oja Unity	200	22	4.4
Oja Ipata	200	11	2.2
Ready-to-eat gizzard	100	10	2
Total	500	43	8.6

**Table 3:** *Salmonella* species isolated from different breeds of chickens in the Ilorin metropolis.

Breed	Oja Unity	Oja Ipata	Oja Unity Number of positive samples (%)	Oja Ipata Number of positive samples (%)	Total for layers and broilers
Layers (*n* = 218)	92	126	10 (4.59)	6 (2.75)	7.3%
Broilers (*n* = 164)	98	66	12 (7.32)	5 (3.1)	10.4%
Cockerels (*n* = 10)	6	4	—	—	—
Local chicken (*n* = 8)	4	4	—	—	—
Total (*n* = 400)	200	200	22 (11.91)	11 (5.85)	17.7%

**Table 4:** Antimicrobial resistance pattern of *Salmonella* species isolated from intestinal contents and ready-to-eat chicken gizzards.

Antimicrobial (*n* = 43)	Resistance	Sensitivity	Symbol	CLIS zone		
				R	I	S
Enrofloxacin	—	43 (100%)	ENR	≤12	13-16	≥17
Nalidixic acid	19 (44%)	24 (56%)	NA	≤15	—	≥17
Gentamycin	12 (28%)	31 (72%)	GN	≤12	13-14	≥15
Ciprofloxacin	43 (100%)	—	CIP	≤15	16-20	≥21
Tetracycline	33 (77%)	10 (23%)	TE	≤14	15-16	≥17
Sulfamethoxazole	29 (67%)	14 (33%)	RL	≤10	11-15	≥16
Ampicillin	43 (100%)	—	AMP	≤28	—	≥29
Ceftazidime	43 (100%)	—	CAZ	≤14	15-17	≥18
Cloxacillin	17 (81%)	4 (19%)	OB	≤10	11-12	≥13
Streptomycin	11 (26%)	32 (74%)	S	≤10	11-12	≥13

**Table 5:** Multidrug resistance pattern of *Salmonella* species isolated from intestinal contents and ready-to-eat chicken gizzards.

No.	Antibiotic combination	Antibiotic groups	No. of isolates
3	CPR, CAZ, AMP	Quinone, cephalo, B-lactam	2
4	CPR, TE, CAZ, AMP	Quinone, cycline, cephalo	1
4	CPR, CAZ, AMP, OBS	Quinolone, cephalo, sulphona	2
4	CPR, RL, CAZ, AMP	Quinolone, B-lactam, cephalo	2
5	NA, CPR, TE, RL, CAZ	Quinolone, cycline, B-lactam, cephalo	1
5	CPR, TE, CAZ, AMP, OBS	Quinolone, cycline, cephalo, B-lactam, sulphona	1
5	NA, CPR, CAZ, AMP, OBS	Quinolone, cephalo, B-lactam, sulphona	2
5	CPR, RL, CAZ, AMP, OBS	Quinolone, B-lactam, cephalo, sulphona	2
5	NA, CPR, TE, CAZ, AMP	Quinolone, cycline, cephalo, B-lactam	2
5	CPR, TE, RL, CAZ, AMP	Quinolone, cycline, B-lactam, cephalo	1
6	NA, CPR, S, CAZ, AMP, OBS	Quinolone, aminogly, cephalo, B-lactam, sulphona	1
6	NA, CPR, TE, CAZ, AMP, OBS	Quinolone, cycline, cephalo, B-lactam, sulphona	1
6	CPR, TE, RL, CAZ, AMP, OBS	Quinolone, cycline, cephalo, B-lactam, sulphona	3
6	CPR, TE, S, RL, CAZ, AMP	Quinolone, cycline, aminogly, cephalo, B-lactam	1
6	GN, CPR, TE, S, CAZ, AMP	Quinolone, aminogly, cycline, cephalo, B-lactam	1
6	NA, CPR, TE, S, CAZ, AMP	Quinolone, cycline, aminogly, cephalo, B-lactam	1
6	NA, CPR, TE, RL, CAZ, AMP	Quinolone, cycline, B-lactam, cephalo	2
6	GN, CPR, TE, RL, CAZ, AMP	Quinolone, aminogly, cycline, B-lactam, cephalo	4
6	NA, CPR, S, RL, CAZ, AMP	Quinolone, aminogly, B-lactam, cephalo	1
6	CPR, TE, S, CAZ, AMP, OBS	Quinolone, cycline, cephalo, B-lactam, sulphona	1
7	GN, CPR, S, RL, CAZ, AMP, OBS	Quinolone, aminogly, B-lactam, cephalo, sulphona	1
7	NA, CPR, TE, RL, CAZ, AMP, OBS	Quinolone, cycline, B-lactam, cephalo, sulphona	2
7	NA, GN, CPR, TE, S, CAZ, AMP	Quinolone, aminogly, cycline, cephalo, B-lactam	1
7	NA, CPR, TE, S, RL, CAZ, AMP	Quinolone, cycline, aminogly, B-lactam, cephalo	2
7	NA, GN, CPR, TE, RL, CAZ, AMP	Quinolone, aminogly, cycline, B-lactam, cephalo	1
7	CPR, TE, S, RL, CAZ, AMP, OBS	Quinolone, cycline, aminogly, B-lactam, cephalo, sulphona	1
8	NA, GN, CPR, TE, S, RL, CAZ, AMP	Quinolone, aminogly, cycline, B-lactam, cephalo	3

B-lactams (ampicillin (AMP), cloxacillin (RL)), cephalosporins (ceftazidime (CAZ)), quinolones (nalidixic acid (NA), ciprofloxacin (CPR)), aminoglycosides (gentamycin (GN), streptomycin (S)), cycline (tetracycline (TE)), sulphonamides (sulphonamides (OBS)).

**Table 6:** Occurrence of *Salmonella* serotypes from intestinal contents and ready-to-eat chicken gizzards from the Ilorin metropolis.

*Salmonella* serovars	Number	% from location of sample collection
S. *enterica* serovar *enterica* 45: d: 1, 7	16	37.21 (11 from intestinal contents and 5 from gizzards)
*S.* Haifa	5	11.63 (5 from intestinal contents)
No growth after enriched broth	11	25.58
Contaminated with *Proteus* which could not be serotyped	11	25.58
Total number	43	100

**Table 7:** The presence of virulence genes from *Salmonella* serovars from the Ilorin metropolis.

Location of the samples	*invA*	*stn*
Intestinal contents *Salmonella enterica* subsp. *enterica* serovar 45: d: 1, 7 (*n* = 11) *S.* Haifa (*n* = 5)	16	16
Chicken gizzards *Salmonella enterica* subsp. *enterica* serovar 45: d: 1, 7 (*n* = 5)	5	5
Total	21	21

## Data Availability

The data are available for research and other educational uses.
